# P2X4 receptor modulates gut inflammation and favours microbial homeostasis in colitis

**DOI:** 10.1002/ctm2.1227

**Published:** 2023-04-21

**Authors:** Peijie Zhong, Hang Wu, Yuanqiao Ma, Xiaoxiao Xu, Yizhuo Jiang, Chaolei Jin, Qiaozhen Zhu, Xinlei Liu, Zhimin Suo, Junpeng Wang

**Affiliations:** ^1^ Infection and Immunity Institute and Translational Medical Center, Huaihe Hospital Henan University Kaifeng China

**Keywords:** gut microbiota, gut permeability, inflammatory bowel diseases, P2X4 receptor

## Abstract

**Background:**

Inflammatory bowel disease (IBD) is a non‐specific chronic inflammatory disease of the intestine. In addition to genetic susceptibility, environmental factors and dysregulated host immunity, the gut microbiota is implicated in the pathogenesis of Crohn's disease (CD) or ulcerative colitis (UC), the two primary types of IBD. The P2X4 receptor has been demonstrated to have a crucial role in preventing infection, inflammation, and organ damage. However, it remains unclear whether the P2X4 receptor affects IBD and the underlying mechanisms.

**Methods:**

Colitis was induced in mice administrated with dextran sodium sulphate (DSS). 16S rDNA sequencing was used to analyze the gut microbiota in knockout and wild‐type mice. Clinical and histopathological parameters were monitored throughout the disease progression.

**Results:**

Gene Expression Omnibus analysis showed the downregulation of *P2RX4 (P2rx4)* expression in colonic tissues from patients or mice with IBD. However, its expression at the protein levels was upregulated on day 4 or 6 and then downregulated on day 7 in C57BL/6 mice treated with DSS. Gene ablation of *P2rx4* aggravated DSS‐induced colitis accompanying gut microbiota dysbiosis in mice. Moreover, P2X4 receptor‐positive modulator ivermectin alleviated colitis and corrected dysregulated microbiota in wild‐type C57BL/6 mice. Further antibiotic‐treated gut microbiota depletion, cohousing experiment, and fecal microbiota transplantation proved that gut microbiota dysbiosis was associated with the aggravation of colitis in the mouse model initiated by *P2rx4*.

**Conclusions:**

Our findings elaborate on an unrevealed etiopathophysiological mechanism by which microbiota dysbiosis induced by the P2X4 receptor influences the development of colitis, indicating that the P2X4 receptor represents a promising target for treating patients with CD and UC.

## Graphical abstract

Inactivated P2X4 receptor worsens the severity of dextran sodium sulphate‐induced colitis in mice, which is associated with gut inflammation, permeability, and microbiota dysbiosis.

Ivermectin, an allosteric P2X4 receptor activator, reduces colitis and restores dysregulated microbiota in mice.

The P2X4 receptor is a potential target for combating inflammatory bowel diseases in Crohn's disease and ulcerative colitis.

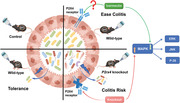

## 1 INTRODUCTION

Crohn's disease (CD) and ulcerative colitis (UC) are chronic, recurring intestinal diseases of unknown aetiology, and are the two main clinical phenotypes of inflammatory bowel disease (IBD).^1^ Proinflammatory mediators, such as tumour necrosis factor (TNF)‐α produced by immune cells in the intestine, are associated with the severity of IBD.^2^ In addition to genetic susceptibility, environmental factors, and a dysregulated host immune system, intestinal microbial dysbiosis has been linked to the relapse in refractory IBD and implicated in an IBD feature.^2^, ^3^, ^4^

Adenosine triphosphate (ATP) is a major source of energy for life and an inflammatory mediator in the immune system.^5^, ^6^ Additionally, it can cause a wide range of responses in cells that have a variety of purinergic receptors.^7^, ^8^ The P2 receptor family, which includes the ionotropic receptor P2X and the G protein‐coupled receptor P2Y, is the family of ATP‐activated purinergic receptors.^9^, ^10^ Currently, seven P2X receptors have been cloned as P2X1‐P2X7.^11^

The P2 receptor family is widely distributed and expressed in the enteric nervous system and epithelial cells, suggesting that it has a key role in IBD pathogenesis. Mice lacking *P2ry6* are more susceptible to colitis induced by dextran sodium sulphate (DSS) via ulcer promotion, suggesting a protective role in IBD.^12^

Furthermore, P2X7 receptor expression varies in the mucosa of patients with active and quiescent IBD. Its inactivation may impair inflammatory response but increase tumor incidence in DSS‐induced colitis‐associated cancer, as it is involved in the processing and release of interleukin (IL)‐1β.^13^, ^14^ Therefore, these findings suggest that the P2 receptor family may be involved in maintaining intestinal homeostasis. It is worth noting that the P2X4 receptor has recently been shown to have an immunomodulatory role in preventing infection, inflammation, and organ damage.^15^ In mouse cecum ligation and puncture‐induced sepsis models, P2X4 receptor activation could increase survival while decreasing bacterial load, inflammatory mediators and organ damage.^16^ However, whether activation or inactivation of the P2X4 receptor influences IBD is currently unknown.

As an intricate ecosystem, the gut has a large and diverse microbial community known as the gut microbiota.^17^, ^18^ The human gut microbiota contains probiotics and pathogenic bacteria that coexist harmoniously in a healthy body. However, this balance can be disrupted by abnormal environmental factors and genetic variations. The pathogenic bacteria will colonize and grow in the gut following gut microbiota dysbiosis before moving on to the host's other organs outside the intestinal tract since the change in intestinal mucosal permeability. Significantly, probiotics in the gut can be transformed into pathogenic bacteria via gene transfer.^19^ A dysregulated gut microbiota alters the intestinal microenvironment and damages intestinal mucosal cells, driven by gut microbiota dysbiosis and inflammatory pathway activation mediated by the inflammatory microenvironment. IBD is a multifactorial disease caused by genetic variations, gut microbiota dysbiosis and abnormal environmental mediators.^20^, ^21^ Therefore, targeting gut microbiota has emerged as the most effective and ideal approach for preventing and treating IBD. The P2 receptor family has also been implicated in regulating the gut microbiota.^22^ For instance, the P2X1 receptor has been shown to reduce inflammation in colitis, possibly via balancing gut microbiota.^23^ However, it is still unclear whether the P2X4 receptor can influence colitis by modulating the gut microbiota balance.

In the present study, we used a colitis model induced by DSS in mice to identify the potential effect of the P2X4 receptors on IBD and further explored its interaction with microbiota‐associated immunity. We observed that *P2rx4* gene ablation exacerbated colitis while activation attenuated it in mice, which involved the alteration of inflammation, gut microbiota dysbiosis, and intestinal mucosal permeability. Thus, the P2X4 receptor could be a promising new target for the progression of potential IBD therapeutic strategies.

## 2 MATERIALS AND METHODS

### 2.1 Animals

Cyagen Biosciences used the CRISPR/Cas9 system to create *P2rx4^−/−^* mice on a C57BL/6 background*. P2rx4* ^−/+^ heterozygous mice were bred in cages, and their offspring genotypes were determined by polymerase chain reaction (PCR) (Table S1 and Figure S1). Finally, mice with *P2rx4* ^+/+^ (wild‐type, WT), *P2rx4* ^−/+^ (heterozygous deletion) and *P2rx4* ^−/−^ (homozygous deletion) were obtained. *P2rx4* ^−/−^, *P2rx4* ^−/+^ and WT littermates had no discernible differences in utero selective fertilization/mortality, development, or growth. Additionally, Beijing SPF Biotechnology Co., Ltd (China) provided other female WT mice, aged 8–10 weeks, for the study of ivermectin's effect in DSS‐induced IBD. All mice were freely fed with regular water and basic feed under the condition of 12 h of light alternating between day and night. The Ethical Committee of Huaihe Hospital of Henan University confirmed that all experimental procedures were carried out following the NIH's guidelines for using experimental animals.

### 2.2 DSS‐induced colitis model

To establish the colitis model, female WT, *P2rx4* ^−/+^and *P2rx4* ^−/−^ mice at 8–10 weeks of age were given DSS at a dose of 3% (w/v) (MW = 36 000–50 000; Cat No CD4421, Coolaber, Beijing, China) for free drinking for 6 days, followed by 1 day of regular water. Body weight was monitored daily in mice throughout the study. The disease activity index (DAI), as previously stated,^24^ was utilized to determine the colitis severity. After mice were euthanized with CO_2_ on day 7, the entire colorectum was resected, and its length was measured.

### 2.3 Histopathology

On day 7, the colons were collected and cleansed of all faeces before being rolled into a Swiss roll and immersed in neutral buffered formalin. Sections of Swiss rolls were stained with hematoxylin and eosin (H&E), or Periodic Acid‐Schiff (PAS). The histopathological images were captured under a light microscope (Nikon Ti2‐E, Tokyo, Japan). Two trained researchers independently assessed histological scores based on previous study criteria.^24^

### 2.4 Colon tissue supernatant and enzyme‐linked immunosorbent assay

Following the preparation of homogenates from colonic tissues in tissue lysate buffer, the supernatant was obtained to determine the concentrations of proinflammatory cytokines such as IL‐1β (Cat No DY401, R&D Systems Inc., Minnesota, USA), IL‐6 (Cat No 88‐7064‐76, eBiosciences, San Diego, CA, USA), interferon (IFN)‐γ (Cat No 551866) and TNF‐α (Cat No 558534) (Both from BD biosciences, San Diego, CA, USA) using corresponding enzyme‐linked immunosorbent assay (ELISA) kits. We used Bradford Protein Assay (BCA) Kit (Cat No PC0020, Solarbio, Beijing, China) to access the protein concentrations in samples.

### 2.5 Western blotting

We used RIPA lysis buffer (Cat No R0010, Solarbio, Beijing, China) containing PMSF (Cat No P0100, Solarbio) and phosphatase inhibitors (Cat No G2007, Servicebio, Wuhan, China) to extract total colonic protein. The protein concentrations of each sample were determined using a BCA kit. After the same amount of protein was resolved on SDS‐PAGE gel and transferred to nitrocellulose membranes. The membrane was blocked with 5% non‐fat milk prior to being incubated with the following primary antibodies for the following proteins: phospho‐p38 (p‐p38, Clone D3F9, Cat No 4511, 1:1000), p38 (Clone D13E1, Cat No 8690, 1:1000), phospho‐ERK (P‐ERK, Clone L34F12, Cat No 4696, 1:1000), ERK (Clone 137F5, Cat No 4695, 1:1000), phospho‐JNK (P‐JNK, Clone G9, Cat No 9255, 1:1000), JNK (Cat No 9252, 1:1000) (all from Cell Signaling Technologies, Danvers, MA, USA), β‐actin (clone AC‐15, Cat No A5441, 1:10 000, Sigma‐Aldrich, USA), P2X4 (Clone 1A5A6, Cat No 66416‐1‐Ig, 1:5000), and GAPDH (Clone 1E6D9, Cat No 60004‐1‐Ig, 1:50 000) (both from Proteintech, Rosemont, IL, USA). After that, the membrane was incubated with a horseradish peroxidase‐conjugated secondary antibody (Cell Signaling Technologies), and then we visualized the protein bands using enhanced chemiluminescent reagents (Millipore, Burlington, MA, USA). ImageJ software was used for quantitative analysis.

### 2.6 Bacterial translocation evaluation

On day 7, after mice treated with DSS were euthanized with CO_2_, mesenteric and inguinal lymph nodes and spleen were harvested to assess bacterial translocation. The above tissues were ground, crushed, and filtered in cold saline to obtain the supernatant. The supernatant was inoculated on Luria‐Bertani solid medium (Tryptone 10 g/L, Yeast Extract 5 g/L, NaCl 10 g/L, Agar 15 g/L) to obtain Colony forming units. The migration rate was obtained following the formula: (the number of plates growing colonies in the group/the total number of samples in the group) × 100%.

### 2.7 Permeability assay using Evans blue in vitro

On day 7, mice treated with DSS were euthanized with CO_2_ and then the luminal contents were cleaned with sterile, cold PBS before the two ends of the luminal tube were ligated to prepare the colorectal intestinal sac described as previously.^25^ The colorectal intestinal sac was then injected with 100 μl of Evans blue (EB) solution (Cat No E2129, Sigma‐Aldrich) [1.5% (w/v) in PBS] and placed in 20 ml of Krebs solution (Sigma‐Aldrich). After 30 min, the colorectal intestinal sac was washed with PBS until the flushing solution became clear, dried for 24 h at 37°C, and obtained a dry weight. It was then cultured at 55°C for another 24 h with 1 ml of formamide (Sigma‐Aldrich). Finally, the intestinal colorectal sac was removed, and the supernatant was centrifuged. The absorbance was measured at 655 nm.

### 2.8 16S rDNA sequencing

Following a published protocol,^26^ fresh cecal contents were mechanically (bead‐beating) disrupted, and total genomic DNA was purified using phenol/chloroform. PCR was used to amplify the V3‐V4 sections of the 16S rDNA genes using the specific paired primers (338F, 5′‐ACTCCTACGGGAGGCAGCA‐3′) and 806R (5′‐GGACTACHVGGGTWTCTAAT‐3′). We purify and recovered the amplified product using magnetic beads (VAHTS DNA Clean Beads; Vazyme Biotech Co., Ltd, China). The recovered products were amplified using PCR and quantified by fluorescence. The samples were mixed in proportion to the sequencing requirements of each sample, based on the quantitative fluorescence results. The sequencing library was constructed using Illumina TruSeq Nano DNA LT Library Prep Kit and then the sequencing was conducted on the Illumina NovaSeq platform by Suzhou PANOMIX Biomedical Tech Co. Ltd.

### 2.9 Gut microbiota analysis

Firstly, the original off‐machine high‐throughput sequencing data were screened for sequence quality, and the problematic samples were retested. The library and samples were divided based on the original sequence index and Barcode information, which was screened for quality, and the Barcode sequence was removed. QIIME2 DADA2 calls DADA2 for quality control, denoising, concatenation, and chimerism removal. Amplicon sequence variants (ASVs) feature sequences and ASV tables were combined after denoising all libraries and singletons ASVs were removed. Then, with a sequence similarity criterion of 100%, we used the SILVA reference database classifier (version 138) to classify ASVs. Alpha and beta diversity were also identified in QIIME 2. Principle coordinates analysis (PCoA), heatmap analysis, unweighted UniFrac similarity clustering and species abundance analysis were all conducted using R version 4.1.3. Differentially abundant genera between groups were analyzed by the linear discriminant analysis (LDA) effect size (LEfSe) analysis (http://huttenhower.sph.harvard.edu/lefse/).

### 2.10 Antibiotic cocktail treatment

During antibiotic feeding, mice were given free access to water with cocktail antibiotics (ampicillin at 1 g/L, metronidazole at 1 g/L, vancomycin at 0.5 g/L and neomycin at 0.5 g/L, all from Coolaber) for 7 days, and 200 μl of antibiotic water were gavage every other day during this period.

### 2.11 Cohousing experiment

For the cohousing experiment, two *P2rx4* ^−/−^ mice and two WT mice in one cage were cohoused at 5 weeks. After mice were cohoused for 5 weeks, they were given 3% DSS for 6 days and then switched to regular water for 1 day before being euthanized.

### 2.12 Faecal microbiota transplantation experiment

To better colonize gut microbiota, recipient mice received a cocktail of antibiotics for 7 days before faecal microbiota transplantation (FMT), resulting in gut microbiota depletion. We collected fresh faeces from WT and *P2rx4* ^−/−^ mice (eight weeks old), and then resuspended them in stroke‐physiological saline solution (Cat No SE131; Shuanghe, China). The suspension at 100 mg/mL was then vigorously mixed for 1 min before being centrifuged at 800 × g for 10 min. Following that, 200 μl of the supernatant obtained by centrifugation was gavaged to recipient mice for 5 days. The mice were then given 3% DSS for 6 days before being switched to regular water for one day before being euthanized.

### 2.13 Ivermectin preparation and administration

We randomly divided C57BL/6 female WT or *P2rx4* ^−/−^ mice into four groups: Control, DSS, DSS+2 mg IVM and DSS+5 mg IVM. Except for regular water in the Control group, mice in the other three groups drank 3% DSS solution continuously for 6 days, then replaced with regular water, and sacrificed mice on the 7th day. Ivermectin (Cat No HY‐15310; MedChemExpress LLC, Shanghai, China) was dissolved in 1% carboxymethyl cellulose sodium (Cat No C304951; Aladdin, Shanghai, China). Mice were administrated with ivermectin orally once a day at doses of 0, 2 or 5 mg/kg body weight from day 0 to day 6 (Figure 9A).

### 2.14 Statistical analysis

All data were expressed as means ± standard error (SEM). All statistical analyses, except the microbiota, were carried out using Prism 8.0 (GraphPad Software, San Diego, CA, USA). We utilized the two‐tailed unpaired Student's t‐test to compare the differences between the two groups and a one‐ or two‐way analysis of variance followed by Tukey's test, to examine data from more than two groups. Significance is indicated as *p < .05.

## 3 RESULTS

### 3.1 Active colitis alters P2X4 receptor expression

**FIGURE 1:**
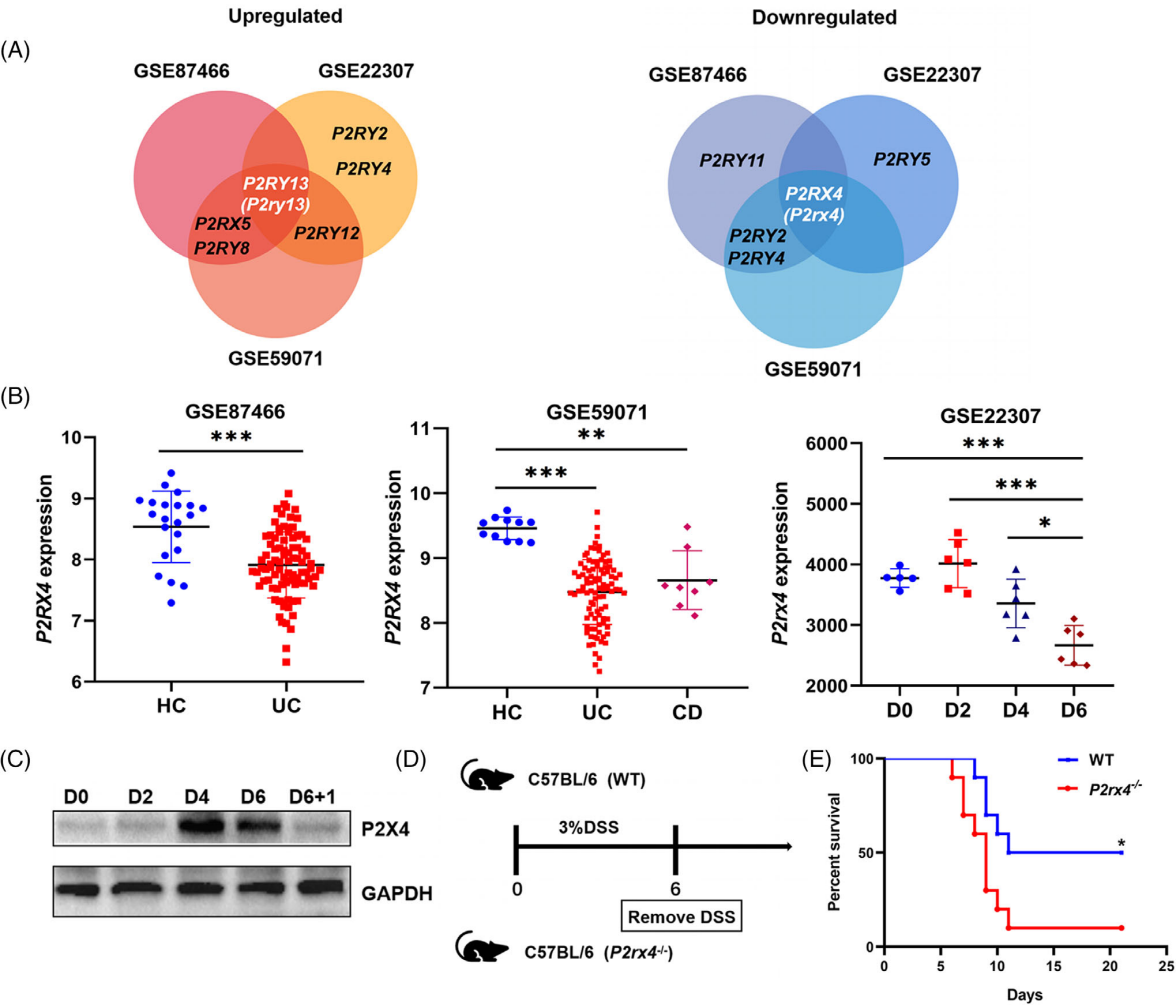
Expression of the purinergic receptor P2X4 is altered in active colitis. (A, B) The intersection of partial purinergic receptors in three datasets from the NCBI Gene Expression Omnibus (GEO) database was analyzed. (GSE87466: active inflammatory bowel disease (IBD) intestinal mucosa expression profiles. Healthy sample, *n* = 21, ulcerative colitis (UC) patients, *n* = 87; GSE59071: active IBD intestinal mucosa expression profiles, Healthy sample, *n* = 11, UC patients, *n* = 97, Crohn's disease (CD) patients, *n* = 8; GSE22307: dextran sodium sulphate (DSS)‐induced murine colitis intestinal tissue expression profile, D0, *n* = 5; D2, D4, D6, *n* = 6, respectively). (C) Western blotting for P2X4 receptor in the colonic epithelium at indicated time points after 3% DSS treatment. (D, E) WT and *P2rx4^−/−^* mice were subjected to 3% DSS for 6 days, and their survival status was tracked till day 21 (*n* = 10/group). Results are expressed as mean ± SEM. *p < .05; **p < .01; ***p < .001.

We wanted to investigate whether the purinergic receptors influence the development of active colitis, we searched and identified three datasets from the Gene Expression Omnibus (GEO) database. Two of them came from patients with active colitis: GSE87466 and GSE59071. GSE22307 was a time course of expression in mouse colon tissue induced by 3% DSS. We primarily accessed the purinergic P2 receptor family changes in active colitis in three datasets. A Venn diagram showed that the P2Y_13_ receptor was upregulated during the development of active colitis, whereas P2X4 receptor expression overlapped at the intersection of purinergic receptors and was downregulated (Figure 1A). Compared to healthy people, patients with active UC or CD had lower levels of *P2RX4* in their inflammatory mucosa (Figure 1B). In the GSE22307 dataset, we discovered that the duration of DSS consumption affected the expression of *P2rx4* in colorectal tissues of DSS‐treated mice. Consistent with this result, *P2rx4* expression was down regulated on day 4 and could be further reduced on day 6 after DSS administration compared to the control group (Figure 1B).

To explore P2X4 receptor protein expression, we collected colorectal tissues from mice administrated with DSS for the indicated time. P2X4 receptor expression was higher on day 4 and 6 after drinking 3% DSS compared to control mice (day 0). P2X4 receptor expression rapidly decreased one day after DSS withdrawal, followed by 1 day with regular water, returning to the pretreatment levels (Figure 1C), suggesting that the P2X4 receptor might be a crucial mediator in acute colitis. To further investigate whether the P2X4 receptor can impact IBD development, we gave *P2rx4^−/−^* mice and WT mice free access to DSS for 6 days, followed by 1 day with regular water (Figure 1D). We found that *P2rx4* gene ablation reduced mouse survival to 10% from 50% in WT mice, indicating P2X4 receptor involvement in IBD (Figure 1E).

### 3.2 *P2r* *x4* ^*/−*^ mice exhibit increased susceptibility to DSS‐induced colitis

**FIGURE 2:**
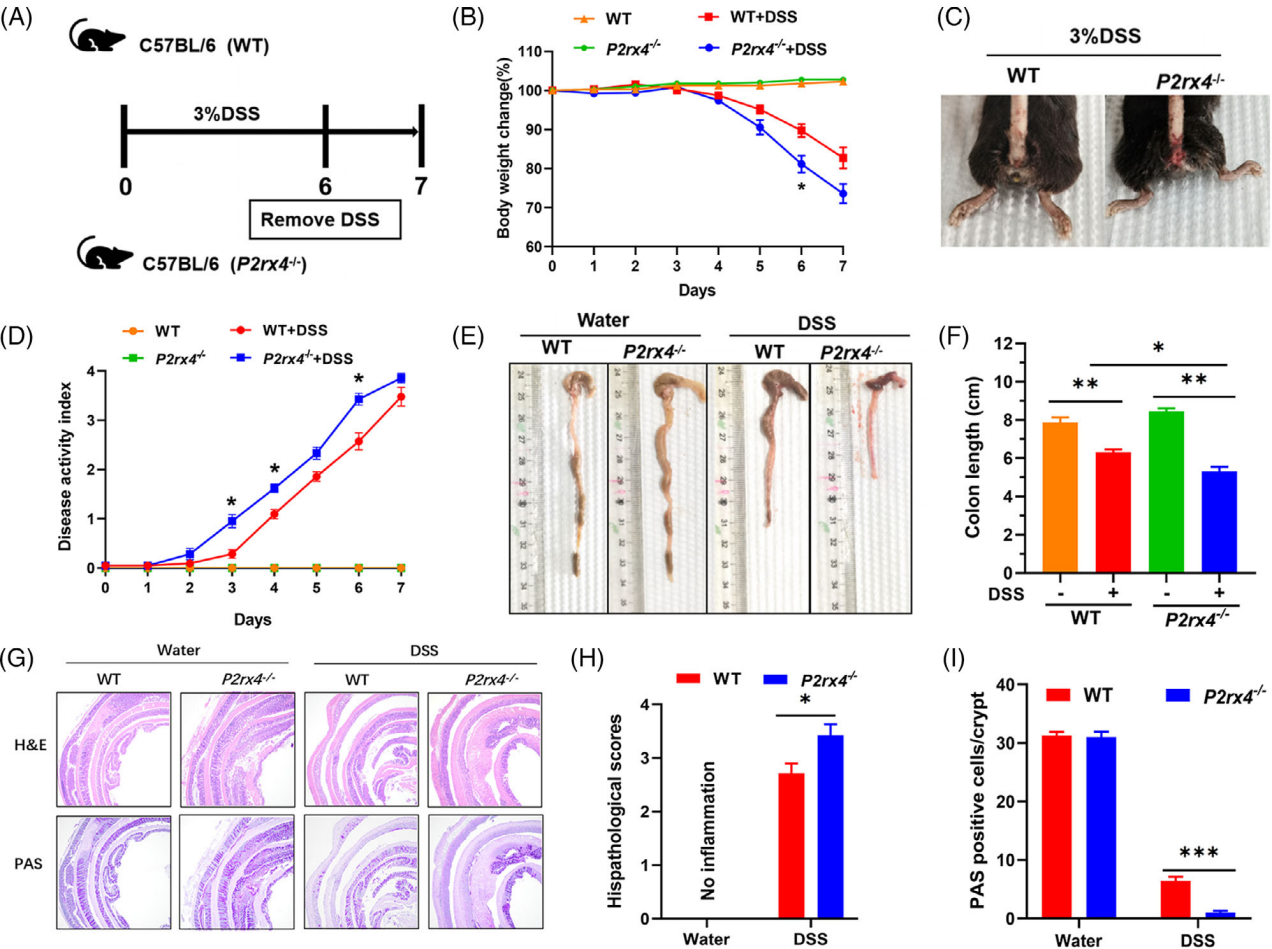
*P2rx4* ablation exhibits increased susceptibility to dextran sodium sulphate (DSS)‐induced colitis. (A) Experimental design of DSS‐induced colitis. See *materials and methods* for details. (B) Body weight change. (C) Representative images of bloody diarrhoea. (D) Disease activity index (DAI) score. (E) Representative pictures of gross colonic appearance. (F) Colon length is shown as a chart. (G) Representative microscopic pictures of hematoxylin and eosin (H&E) staining and Periodic Acid‐Schiff (PAS) staining (original × magnification 40). (H) Changes in colonic section histopathological scores. (I) The number of PAS‐positive cells per crypt. Results are expressed as mean  ±  SEM (B, D, F, H, I). *p*‐Values were calculated using a two‐way analysis of variance (ANOVA) test, *p  <  .05; **p  <  .01. (WT, *n*  =  4; *P2rx4^−/−^*, *n*  =  4; WT+DSS, *n*  =  7; *P2rx4^−/−^* +DSS, *n*  =  7).

To investigate the potential role of the P2X4 receptor in IBD, we first induced acute experimental colitis in *P2rx4^−/−^* and WT mice with free access to 3% DSS for 6 days. We found that loss of *P2rx4* resulted in more severe DSS‐induced colitis than in WT mice (Figure 2). Compared with WT mice treated with DSS, *P2rx4^−/−^* mice had a significantly higher rate of body weight loss and more severe rectal bleeding (Figure 2B,C), resulting in significantly higher DAI scores (Figure 2D). In addition, *P2rx4^−/−^* mice showed more severe colon shortening than WT mice (Figure 2E,F). During colitis, colorectal tissue of *P2rx4^−/−^* mice showed more severely damaged ulcers including more extensive inflammatory cell infiltration and less goblet cells in H&E staining and PAS staining results, respectively (Figure 2G‐I). These findings suggest that *P2rx4* deficiency leads to increased susceptibility to intestinal inflammation. To further rule out the possibility that the P2X4 receptor affects colitis in mice, we administrated DSS to the two different types of mice for six days, then one day with regular water. As shown in Figure S2, we did not observe any difference in body weight loss, DAI, or colon length between them, indicating that the effect of the P2X4 receptor on colitis required the mutation of the two alleles.

### 3.3 *P2rx4* deficiency activates MAPK signaling and exacerbates inflammatory responses

**FIGURE 3:**
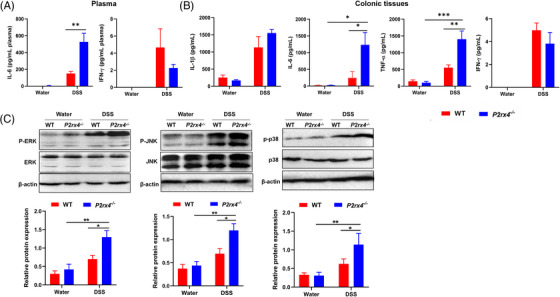
*P2rx4* deficiency activates MAPK signalling and exacerbates inflammatory responses. (A) Interleukin (IL)‐6 and interferon (IFN)‐γ concentrations in the plasma. (B) IL‐6, IL‐1β, IFN‐γ and TNF‐α concentrations in the colonic tissue lysate. (C) Effect of *P2rx4* on MAPK‐related protein expression in mice with colitis. Representative gels were shown, and data were summarized from three independent experiments and analyzed using one‐way analysis of variance (ANOVA) followed by Tukey's test. (WT, *n*  =  4; *P2rx4^−/−^*, *n*  =  4; WT + dextran sodium sulphate (DSS), *n*  =  7; *P2rx4^−/−^* +DSS, *n*  =  7). Results are expressed as mean  ±  SEM. *p  <  .05; **p  <  .01.

Because inflammatory mediators are a driving factor in acute colitis, we evaluated the effects of *P2rx4* deletion on systemic and intestinal inflammatory responses. We found no difference in the production of inflammatory cytokines IL‐1β, IL‐6 and TNF‐α in colonic tissues between *P2rx4^−/−^* and WT mice treated with water. These mice also showed undetectable colonic IFN‐γ, plasma IFN‐γ and IL‐6 levels (Figure 3A). However, DSS treatment could increase the production of these inflammatory cytokines (Figure 3A). *P2rx4^−/−^* mice treated with DSS had higher plasma IL‐6 and colonic IL‐6 and TNF‐α concentrations but not plasma IFN‐γ, colonic IL‐1β or IFN‐γ concentrations than DSS‐induced WT mice.

The MAPK kinase family, including p38‐MAPK, transduces responses to diverse extracellular stimuli.^27^ According to an earlier study, P2X4 receptor‐evoked calcium influx mediates p38MAPK phosphorylation and subsequent downstream activation.^28^ However, the effect of the P2X4 receptor on the MAPK signals in colitis induced by DSS is still unclear. Therefore, we evaluated the expression of MAPK signalling proteins in colorectal tissues from mice with DSS‐induced colitis. We found no difference in the expressions of phosphorylated p38MAPK, ERK and JNK between WT and *P2rx4^−/−^* mice were given regular water. However, DSS treatment upregulated colonic p38MAPK, ERK and JNK phosphorylation, which could be further increased by *P2rx4* gene ablation (Figure 3C), suggesting that increased inflammatory response caused by *P2rx4* deficiency may further enhance MAPK signal activation.

### 3.4 *P2rx4* deficiency increases colonic mucosal permeability in DSS‐induced colitis

**FIGURE 4:**
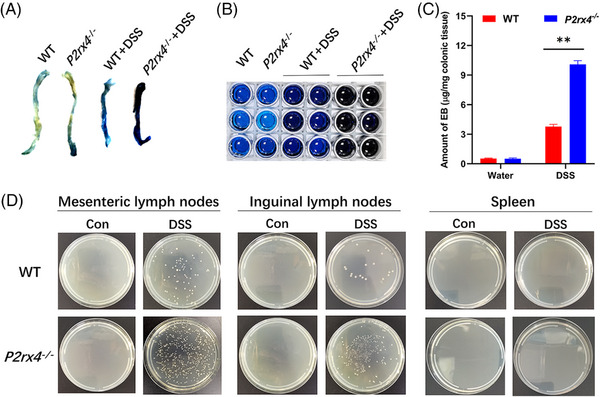
*P2rx4* deficiency affects colonic mucosal permeability and bacterial translocation. (A) The EB exclusion method was used to measure the gut permeability, and the eluded colon was scanned. (B) EB dye eluted from the colon of mice. (C)The amount of EB was measured. Results are expressed as mean  ±  SEM. (D) Representative images of bacteria cultured in mesenteric lymph nodes, inguinal lymph nodes, and spleen. *P < .05, **P < .01. (WT, *n*  =  3; *P2rx4^−/−^*, *n*  =  3; WT + dextran sodium sulphate (DSS), *n*  =  6; *P2rx4^−/−^* +DSS, *n*  =  6). EB, Evans blue.

In animal models of colitis, dysregulated intestinal mucosal permeability may increase susceptibility to colitis.^29^ Therefore, to know whether changes in gut permeability are related to the worsening colitis caused by *P2rx4* deletion, we determined the intestinal mucosal permeability by EB exclusion assay. We did not find any difference in the amount of Evan's blue between *P2rx4^−/−^* and WT mice given regular water. However, DSS administration markedly resulted in an increase of intestinal permeability in DSS‐induced WT mice, which could be exacerbated by *P2rx4* gene ablation (Figure 4A–C).

Furthermore, gut permeability has caused bacterial translocation due to intestinal bacteria leakage. We collected the spleen, and mesenteric and inguinal lymph nodes (LN) to determine the impact of *P2rx4* deletion on bacterial translocation. We found that *P2rx4* deletion significantly increased bacterial translocation to the mesenteric and inguinal LN, followed by a migration rate of 29% (2/7 of WT mice showed bacterial growth with a migration rate of 29%) versus 86% (6/7 of *P2rx4^−/−^* mice showed bacterial growth with a migration rate of 86%) in DSS‐induced WT and *P2rx4^−/−^* mice, respectively (Figure 4D). Mass spectrometry confirmed that the colonies were *Escherichia coli* or *Enterococcus gallinarum* (Figure S3). However, we could not detect the translocation of bacteria in the spleen. These findings suggest that altering gut permeability and bacterial translocation may be involved in the aggravation of colitis caused by deleting *P2rx4*.

### 3.5 Gut microbiota is altered in DSS‐treated *P2rx4* ^*‐/‐*^ mice

**FIGURE 5:**
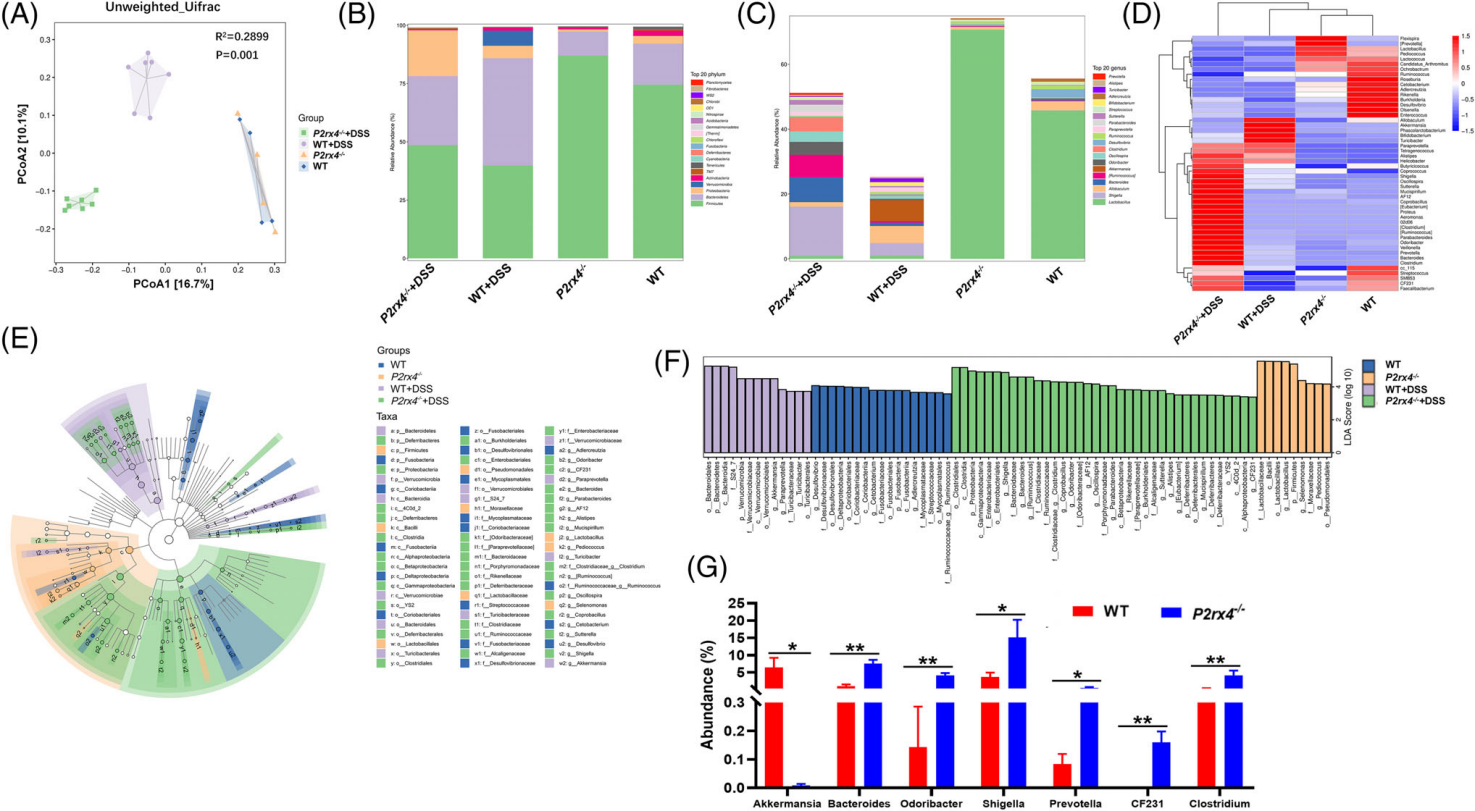
Loss of the P2X4 receptor promotes intestinal dysbiosis. (A) Principle coordinates analysis (PCoA) using unweighted UniFrac distances of beta diversity. (B) Bacterial taxonomic profiling at the phylum level. (C) Bacterial taxonomic profiling at the genus level. (D) Heat map of selected most differentially abundant features at the genus level between WT and *P2rx4^−/−^* mice (E) LEfSe results of each group. (F) Histogram of the LDA scores. (G) Comparison of bacterial species alterations in WT and *P2rx4^−/−^* mice after dextran sodium sulphate (DSS) treatment. (WT, *n*  =  4; *P2rx4^−/−^*, *n*  =  4; WT+DSS, *n*  =  7; *P2rx4^−/−^+*DSS, *n*  =  7).

To define whether gut microbiota profiles are associated with the deterioration of colitis caused by *P2rx4* deletion, we performed a 16S rDNA sequencing in cecal contents. We first accessed the beta diversity using the unweighted UniFrac distance algorithm in PCoA, and we discovered a clear distinction between WT or *P2rx4^−/−^* mice given DSS (Figure 5A). We did not find a difference in the PCoA between WT and *P2rx4^−/−^* mice were given regular water. Further analysis of relative abundance at the phylum level revealed that *P2rx4^−/−^* mice given with DSS had higher abundances of Deferribacteres and Proteobacteria and lower abundances of Bacteroidetes and Verrucomicrobia than in the DSS‐induced WT mice (Figure 5B). Mice given DSS (either WT or *P2rx4^−/−^*) had a lower genus abundance of *Lactobacillus* when compared with mice given regular water. Additionally, compared to *P2rx4^−/−^* mice given DSS, the intestinal flora of DSS‐induced WT mice harboured higher abundances of *Akkermansia* and dampened *Bacteroides*, *Odoribacter*, *CF231*, *Oscillospira*, *Clostridium*, *Prevotella* and *Shigella* (Figure 5C,G). To differentiate the composition of the gut flora between various genotypes, we conducted a comparison heatmap at the family and individual levels according to the ASV abundance (Figure 5D). Utilizing LEfSe and LDA analysis, we assessed the influence of the P2X4 receptor on the gut microbiota composition in the four groups. According to the LDA score, the significantly different strains in the WT and *P2rx4^−/−^* groups given regular water were *Desulfovibrio* and *Lactobacillus*, respectively. Bacteroidales and Clostridiales have considerably changed in the WT and *P2rx4^−/−^* mice following DSS treatment, respectively (Figure 5E,F).

### 3.6 Exacerbation of colitis in *P* *2* *rx* *4* ^*−/−*^ mice depends on the gut microbiota

**FIGURE 6:**
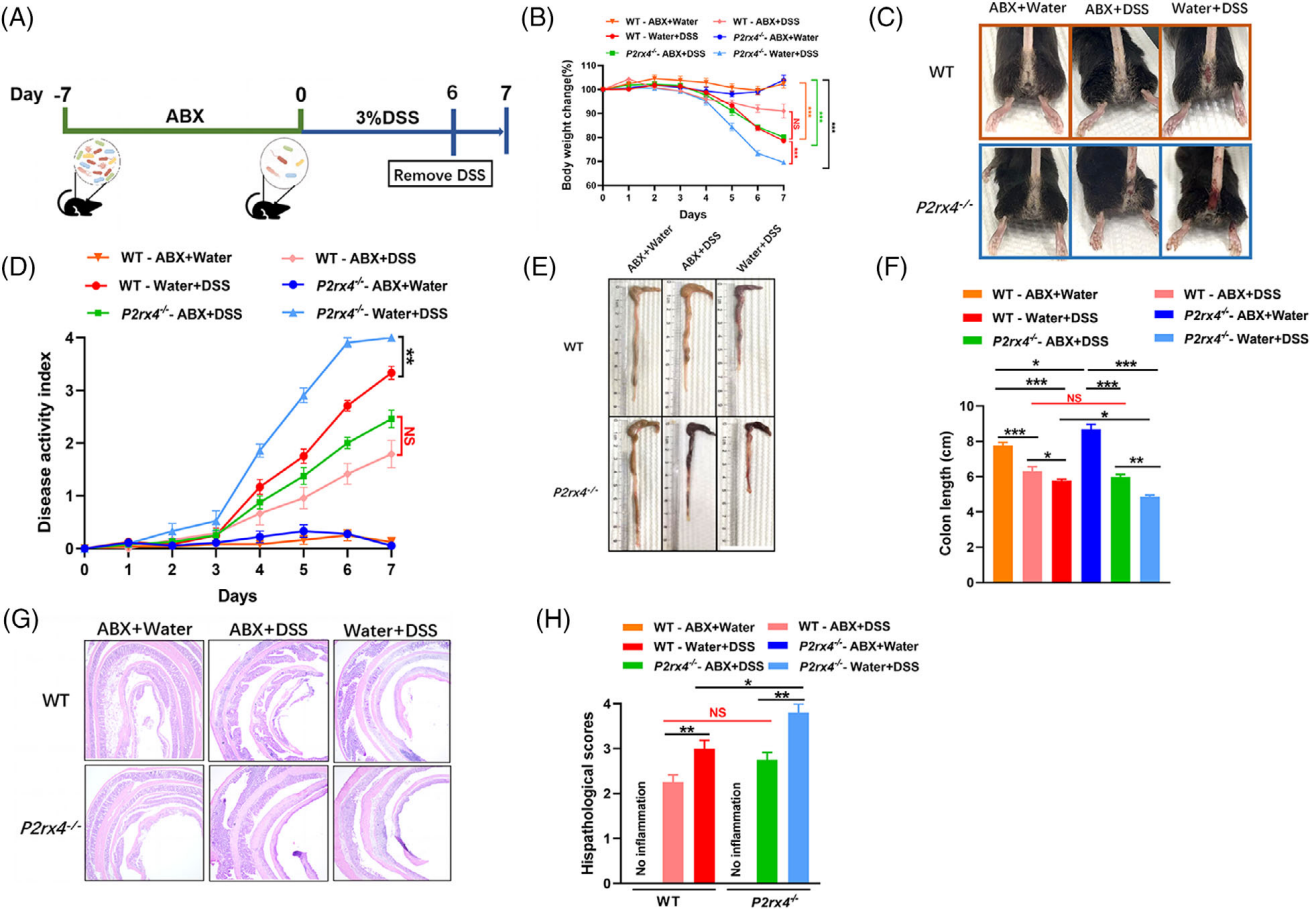
Exacerbation of colitis in *P2rx4^−/−^* mice depends on the gut microbiota. (A) Experimental design of antibiotic cocktail therapy. See *materials and methods* for details. (B) Body weight change. (C) Representative images of bloody diarrhoea. (D) Disease activity index (DAI) score. (E) Representative pictures of the gross colonic appearance. (F) Colon length is shown as a chart. (G) Representative microscopic pictures of hematoxylin and eosin (H&E) staining (original × magnification 40). (H) Changes in colonic section histopathological scores. Results are expressed as mean  ±  SEM (B, D, F, H). *p*‐values were calculated using a two‐way analysis of variance (ANOVA) test, *p  <  .05; **p  <  .01; ***p < .001. (WT ‐ ABX+Water, *n* = 8; *P2rx4^−/−^* ‐ ABX+Water, *n* = 6; WT ‐ ABX+dextran sodium sulphate (DSS), *n* = 8; *P2rx4^−/−^* ‐ ABX+DSS, *n* = 8; WT ‐ Water+DSS, *n* = 8; *P2rx4^−/−^* ‐Water+DSS, *n* = 7).

To further verify whether the deterioration of colitis caused by *P2rx4* deletion is due to gut microbiota dysbiosis, we gave WT and *P2rx4^−/−^* mice an antibiotic cocktail (ABX) to consume their gut microbiota before inducing colitis with DSS (Figure 6A). Compared to mice treated without ABX, both WT and *P2rx4^−/−^* mice treated with ABX showed significantly reduced symptoms of colitis (Figure 6B–F), including weight loss, a lower DAI score, and longer colon length. Importantly, ABX‐treated with DSS *P2rx4^−/−^* mice had body weight loss, DAI scores, colon length, and histological scores that were indistinguishable from ABX‐treated with DSS WT mice (Figure 6B–H), suggesting that the gut microbiota may play a crucial role in the worsening colitis in *P2rx4^−/−^* mice.

### 3.7 The gut microbiota of *P2rx4* ^*−/−*^ mice exacerbates DSS‐induced colitis

**FIGURE 7:**
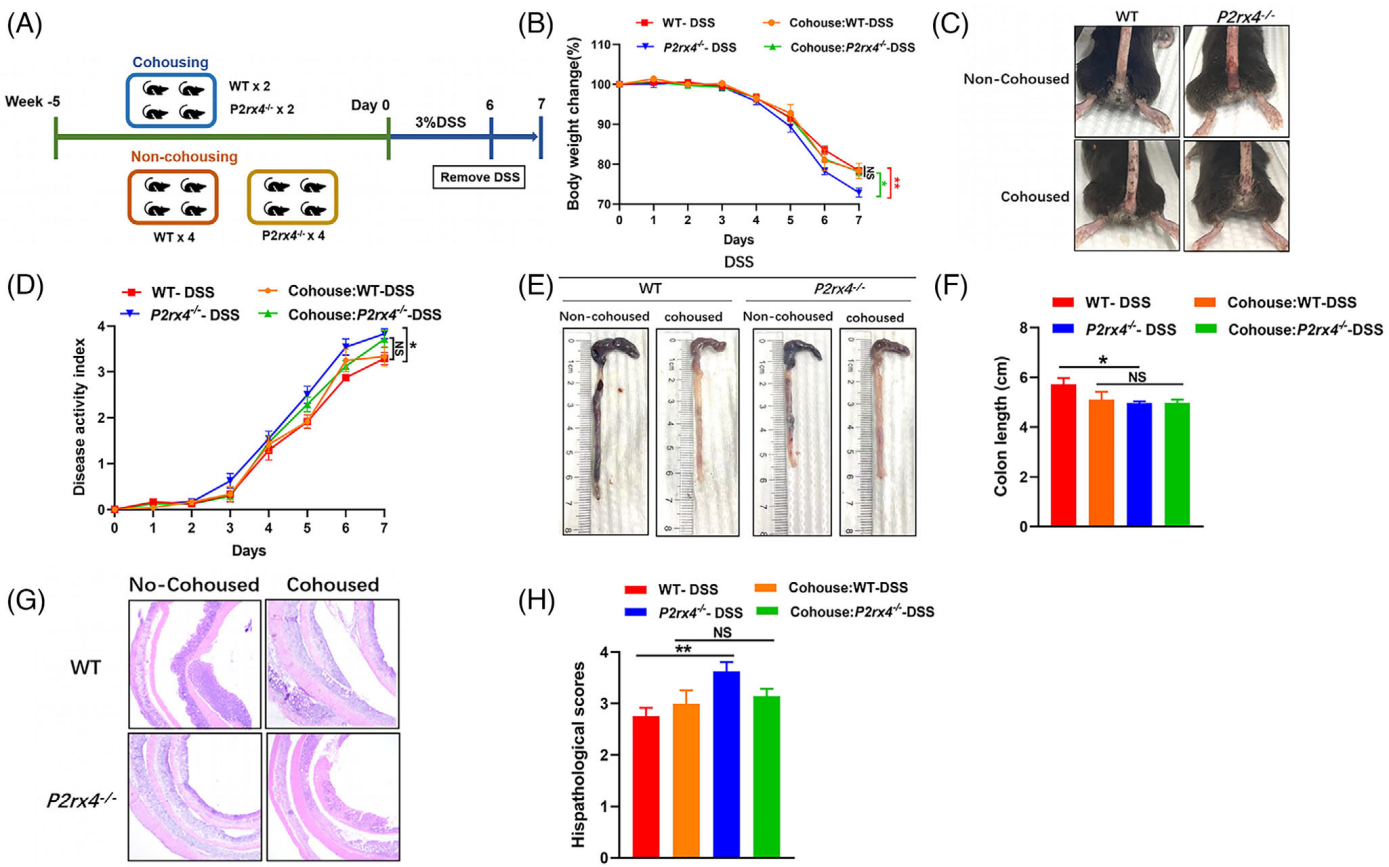
Gut microbiota from *P2rx4^−/−^* mice aggravates dextran sodium sulphate (DSS)‐induced colitis. (A) Design of the cohousing experiment. See *materials and methods* for details. (B) Body weight change. (C) Representative images of bloody diarrhoea. (D) Disease activity index (DAI) score. (E) Representative pictures of the gross colonic appearance. (F) Colon length is shown as a chart. (G) Representative microscopic pictures of hematoxylin and eosin (H&E) staining (original × magnification 40). (H) Changes in colonic section histopathological scores. Results are expressed as mean  ±  SEM (B, D, F, H). *p*‐values were calculated using a two‐way analysis of variance (ANOVA) test, *p  <  .05; **p  <  .01; ***p < .001. (Cohoused: WT, *n* = 8; *P2rx4^−/−^*, *n*  =  8; No‐Cohoused: WT, *n* = 8; *P2rx4^−/−^*, *n*  =  8).

To determine whether a microbiota‐dependent mechanism contributes to counteracting chemical‐induced stress on the gut barrier in *P2rx4^−/−^* mice, we conducted the cohousing experiment (Figure 7A). As shown in Figure 7B, cohoused *P2rx4^−/−^* mice lost less body weight than non‐cohoused *P2rx4^−/−^* mice. Consistent with body weight loss, *P2rx4^−/−^* and WT mice showed similar histopathological changes, colon length, and DAI scores after cohousing (Figure 7C–H).

### 3.8 FMT alleviates colon inflammation in *P2rx4* ^*−/−*^ mice

**FIGURE 8:**
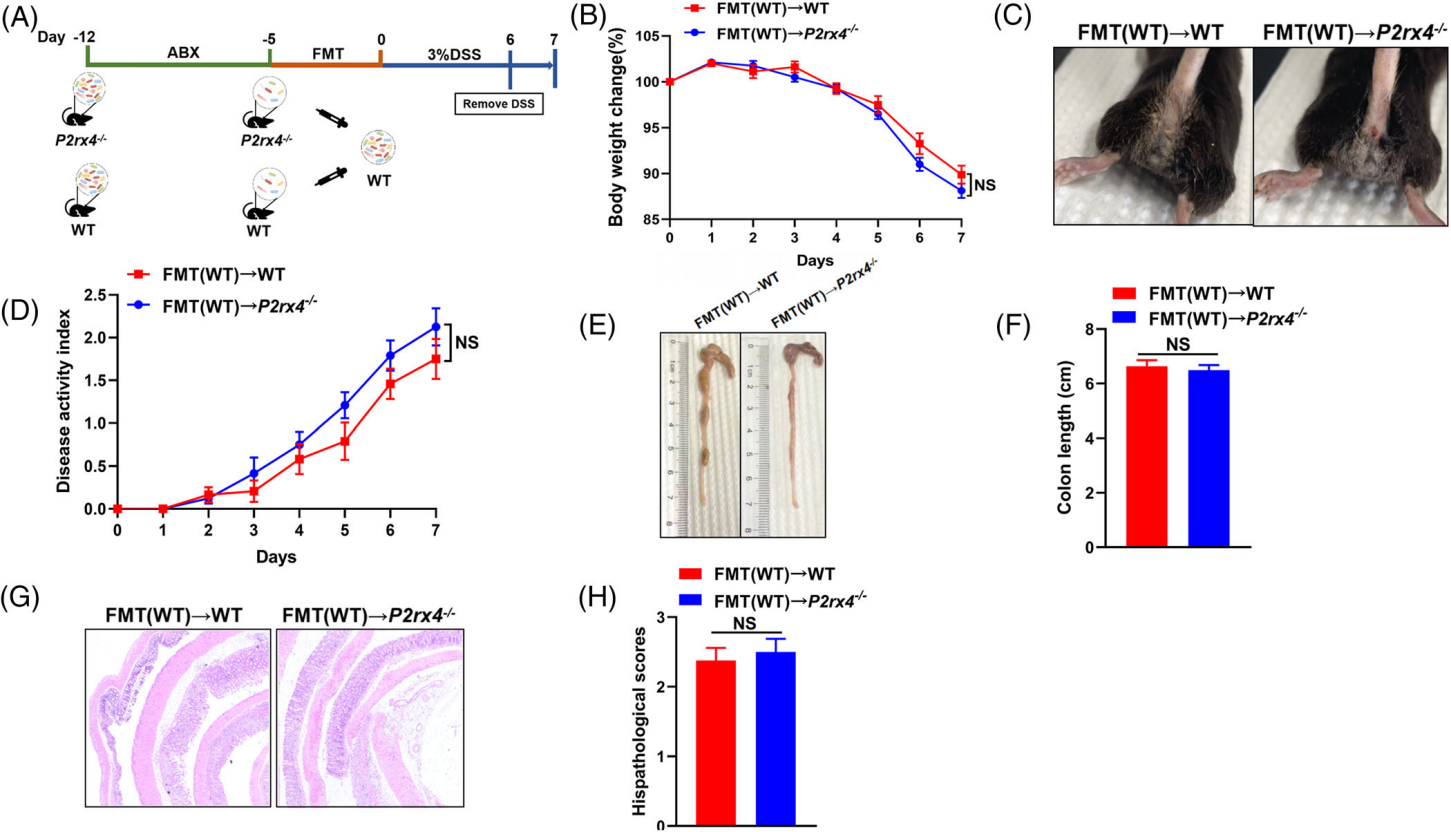
Faecal microbiota transplantation (FMT) alleviates colon inflammation in *P2rx4* ^−/−^ mice. (A) Design of the FMT experiment. See *materials and methods* for details. (B) Body weight change. (C) Representative images of bloody diarrhoea. (D) Disease activity index (DAI) score. (E) Representative pictures of the gross colonic appearance. (F) Colon length is shown as a chart. (G) Representative microscopic pictures of hematoxylin and eosin (H&E) staining (original × magnification 40). (H) Changes in colonic section histopathological scores. Results are expressed as mean  ±  SEM (B, D, F, H). *p*‐values were calculated using a two‐way analysis of variance (ANOVA) test or two‐tailed unpaired Student's t‐test, *p  <  .05; **p  <  .01; ***p < .001. (FMT(WT)→WT, *n*  =  8; FMT(WT)→*P2rx4^−/−^*, *n*  =  8).

Finally, we conducted an FMT test to confirm whether worsening colitis caused by *P2rx4* ablation is mediated by gut microbiota (Figure 8). We found that a significant reduction in weight loss and DAI score was observed in DSS‐treated *P2rx4^−/−^* mice that received the faecal microbiota of WT mice (Figure 8B–D). Furthermore, colonic shortening and colonic epithelial injury induced by DSS were also alleviated in *P2rx4^−/−^* mice, given the gut microbiota of WT mice (Figure 8E–H).

### 3.9 P2X4 receptor potentiation ameliorates DSS‐induced colitis

**FIGURE 9:**
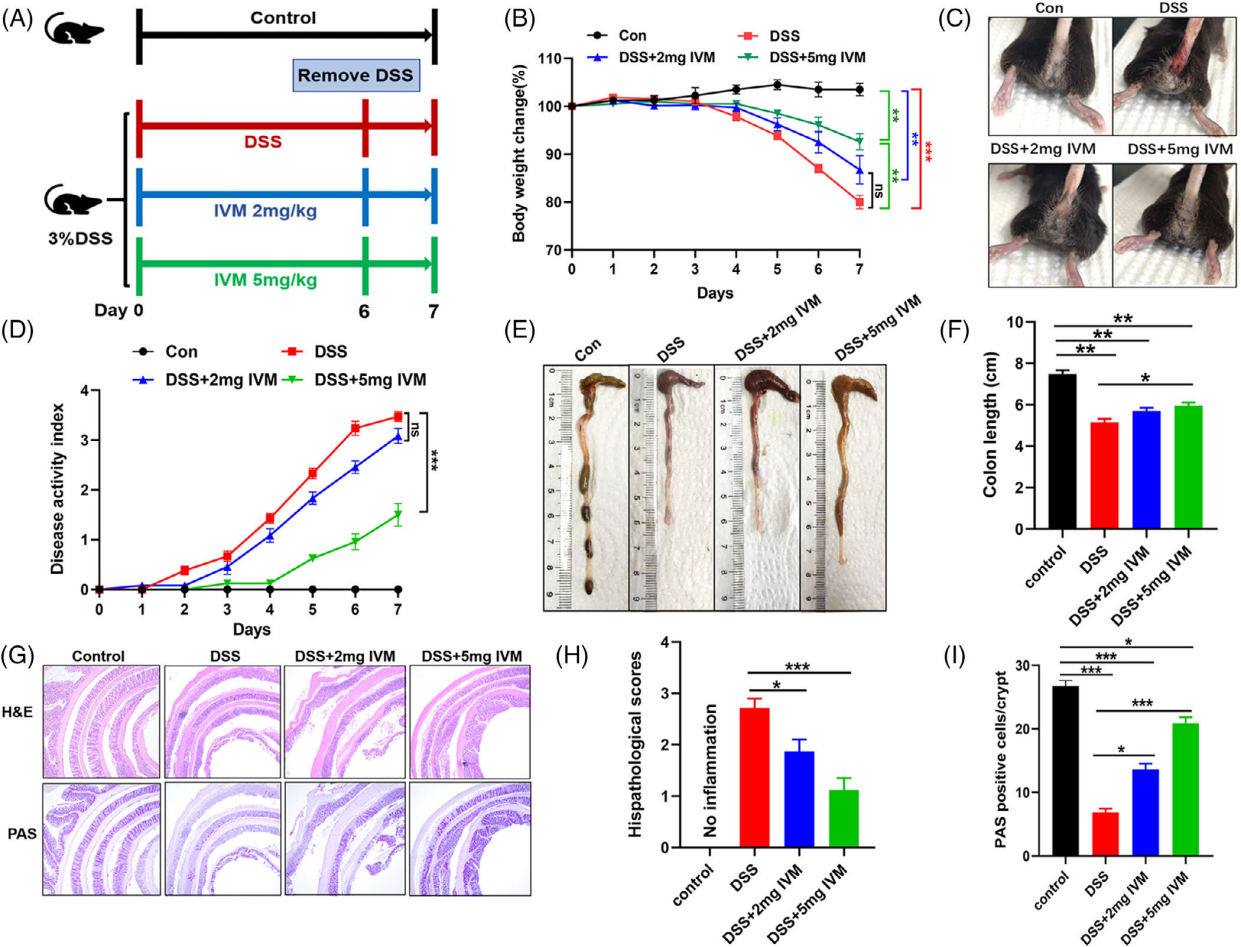
P2X4 receptor potentiation ameliorates dextran sodium sulphate (DSS)‐induced colitis. (A) Experimental design of the role of IVM in colitis in mice. See *materials and methods* for details. (B) Body weight change. (C) Representative images of bloody diarrhea. (D) Disease activity index (DAI) score. (E) Representative pictures of the gross colonic appearance. (F) Colon length is shown as a chart. (G) Representative microscopic pictures of hematoxylin and eosin (H&E) and Periodic Acid‐Schiff (PAS) staining (original × magnification 40). (H) Changes in colonic section histopathological scores. (I) The number of PAS‐positive cells per villus/crypt. Results are expressed as mean  ±  SEM (B, D, F, H, I). *p*‐values were calculated using a two‐way analysis of variance (ANOVA) test, *p  <  .05; **p  <  .01; ***p < .001. (Control, *n*  =  4; DSS, *n*  =  7; DSS+2 mg IVM, *n*  =  8; DSS+5 mg IVM, *n*  =  8). Control, Con.

To investigate the role of P2X4 receptor potentiation in acute colitis, we administered DSS to WT mice in the absence or presence of ivermectin at the indicated doses (Figure 9A). Our findings demonstrated that, when compared with DSS‐treated mice without ivermectin, ivermectin at 5 mg/kg was most effective in protecting against colitis, including reducing body weight loss and increased colon length, as well as lowering DAI scores, which were not present in mice given ivermectin at 2 mg/kg (Figure 9B–F). Furthermore, mice given ivermectin had increased crypts deep, less mononuclear cell infiltration and mucosal damage, and more goblet cells in colon tissue than those in mice given only DSS (Figure 9G–I). Our data indicate that P2X4 receptor activation by ivermectin could attenuate DSS‐induced colitis.

### 3.10 P2X4 receptor potentiation can reduce inflammatory response by impairing the MAPK signals

**FIGURE 10:**
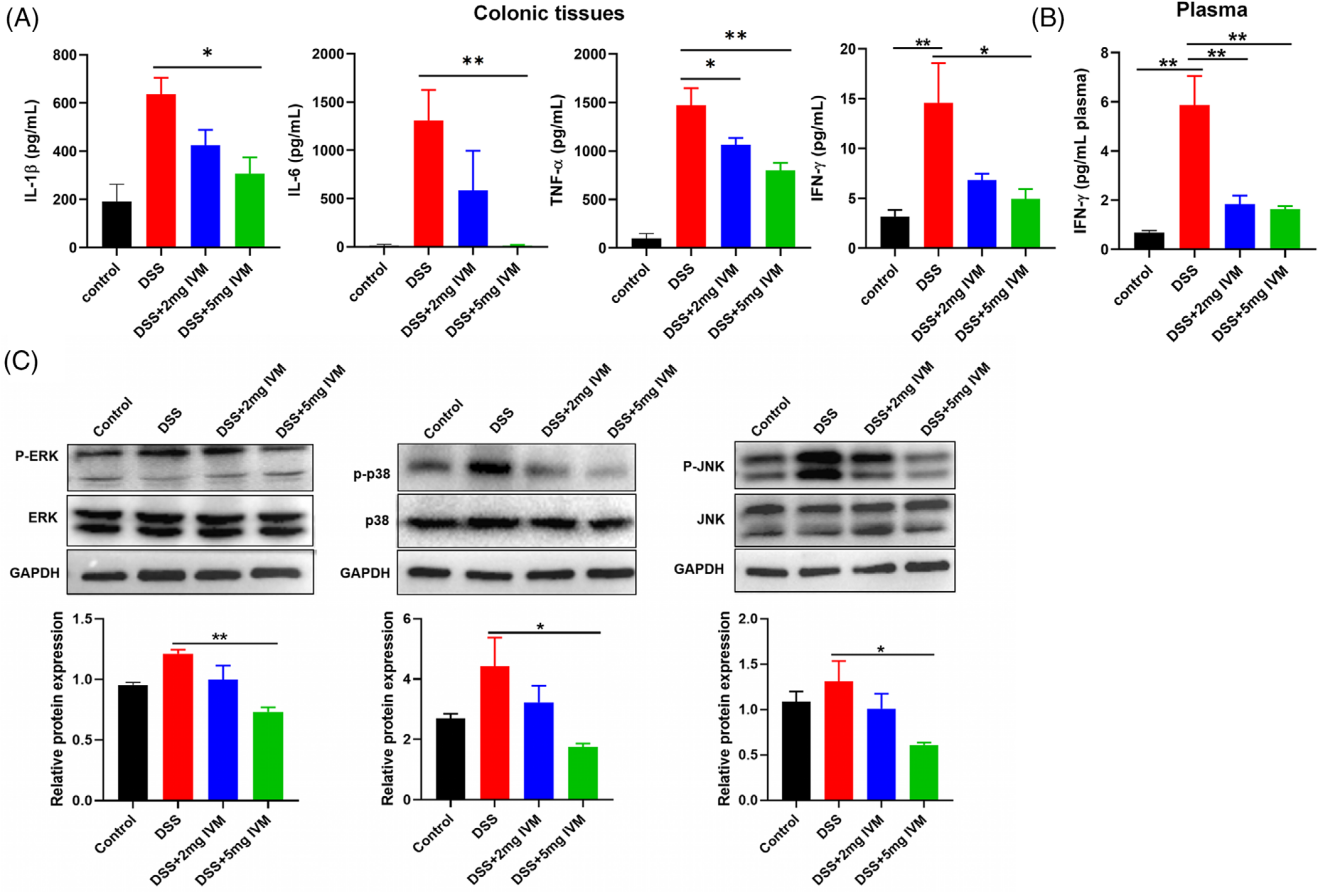
Ivermectin can inhibit the MAPK signalling pathway and reduce inflammation. (A) Interleukin (IL)‐6, IL‐1β, interferon (IFN)‐γ and tumour necrosis factor (TNF)‐α concentrations in the colonic tissue lysate. (B) IFN‐γ concentrations in the plasma. (C) Effect of ivermectin on MAPK pathway‐related protein expression in mice with colitis. Representative gels were shown, and data were summarized from three independent experiments and analyzed using one‐way analysis of variance (ANOVA) followed by Tukey's test. (Control, *n*  =  7; dextran sodium sulphate (DSS), *n*  =  7; DSS+2 mg IVM, *n*  = 7; DSS+5 mg IVM, *n*  =  7). Results are expressed as mean  ±  SEM. *p  <  .05; **p  <  .01.

To verify whether an inflammatory response is involved in ivermectin's protective effect against acute colitis induced by DSS, we used an ELISA assay to measure proinflammatory cytokine levels in colonic tissues and plasma. As shown in Figure 10A,B, compared with mice administrated with DSS, ivermectin treatment reduced these proinflammatory cytokine levels including IL‐1β, IL‐6, TNF‐α and IFN‐γ.

Since MAPK signals are involved in worsening colitis caused by *P2rx4* deficiency, we wanted to test the MAPK‐related protein expression using a Western Blotting assay to that could be impacted by P2X4 receptor activation. When compared to DSS‐treated mice, mice given ivermectin at 5 mg/kg had lower phosphorylation of ERK, JNK and p38MAPK in the colonic tissues (Figure 10C). These findings suggest that P2X4 receptor activation may have therapeutic potential in colitis by reducing inflammatory responses.

### 3.11 Changes in gut microbiota after P2X4 receptor activation

**FIGURE 11:**
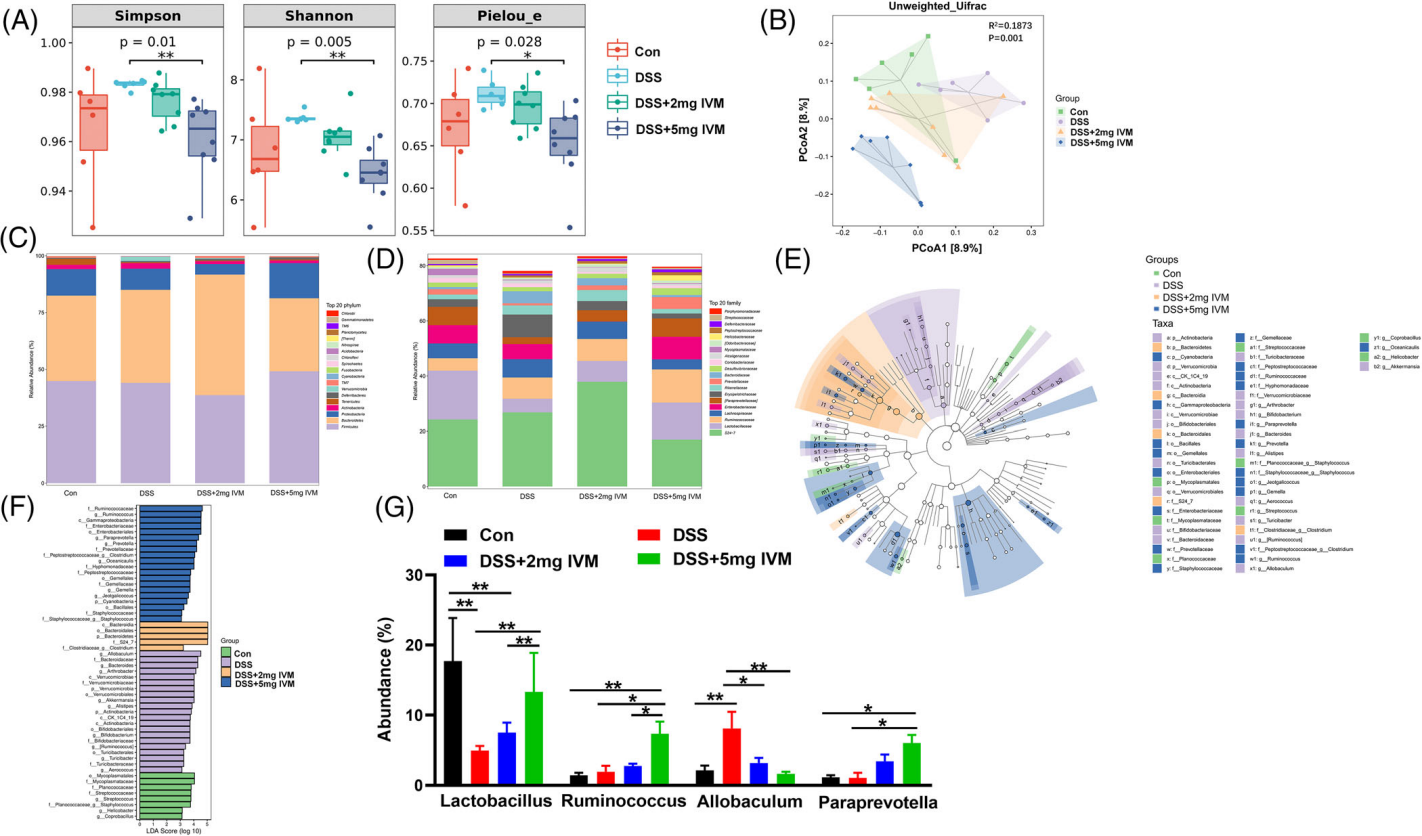
Changes in Gut microbiota after P2X4 receptor activation. (A) Grouping box diagram of alpha diversity index. (B) Principle coordinates analysis (PCoA) using unweighted UniFrac distances of beta diversity. (C) Bacterial taxonomic profiling at the phylum level. (D) Bacterial taxonomic profiling at the family level. (E) linear discriminant analysis (LDA) effect size (LEfSe) results of each group. (F) Histogram of the LDA scores. (G) Comparison of bacterial species alterations after P2X4 receptor activation.  (WT, *n*  =  6; DSS, *n*  =  6; DSS+2 mg IVM, *n*  =  8; DSS+5 mg IVM, *n*  =  8).

To further reveal whether the gut microbiota is involved in ivermectin's protective effect against DSS‐induced colitis, we utilized 16S rDNA sequencing to clarify microbial composition. Figure 11A showed that ivermectin at 5 mg/kg reduced the Simpson, Shannon, and Pielou_e indexes compared to the DSS‐treated group. Unweighted PCoA analysis revealed significant differences between the DSS‐treated group with or without 5 mg/kg ivermectin (Figure 11B). Furthermore, the results revealed that the taxonomic communities of all samples were comparable at the phylum level and that Firmicutes, Bacteroidetes, Proteobacteria, and Actinobacteria were present in comparably high abundance in all samples (Figure 11C). At the genus level, the enrichment of the *Allobaculum* was greatest in the DSS‐treated group, but its abundance in the DSS‐treated groups with 2 and 5 mg/kg ivermectin was comparable to the control group.

The LEfSe analysis showed the hierarchy and abundance of intestinal flora among different mice groups (Figure 11E,F). According to the LDA score, *Ruminococcus* was more abundant in the control group's gut microbiota, whereas *Allobaculum* was enriched in the DSS‐only group. Furthermore, we found that DSS‐induced mice given ivermectin at 2 or 5 mg/kg had a high genus abundance of the *Lactobacillus, Ruminococcus* and *Paraprevotella* (Figure 11D–G). Bacteroides or Ruminococcaceae were the most abundant microbes in DSS‐induced mice given ivermectin at 2 or 5 mg/kg, respectively (Figure 11E,F). These findings aligned with the abundance of phylum abundance of gut microbiota.

### 3.12 Effect of ivermectin on DSS‐induced colitis in *P2rx4* ^*−/−*^ mice

**FIGURE 12:**
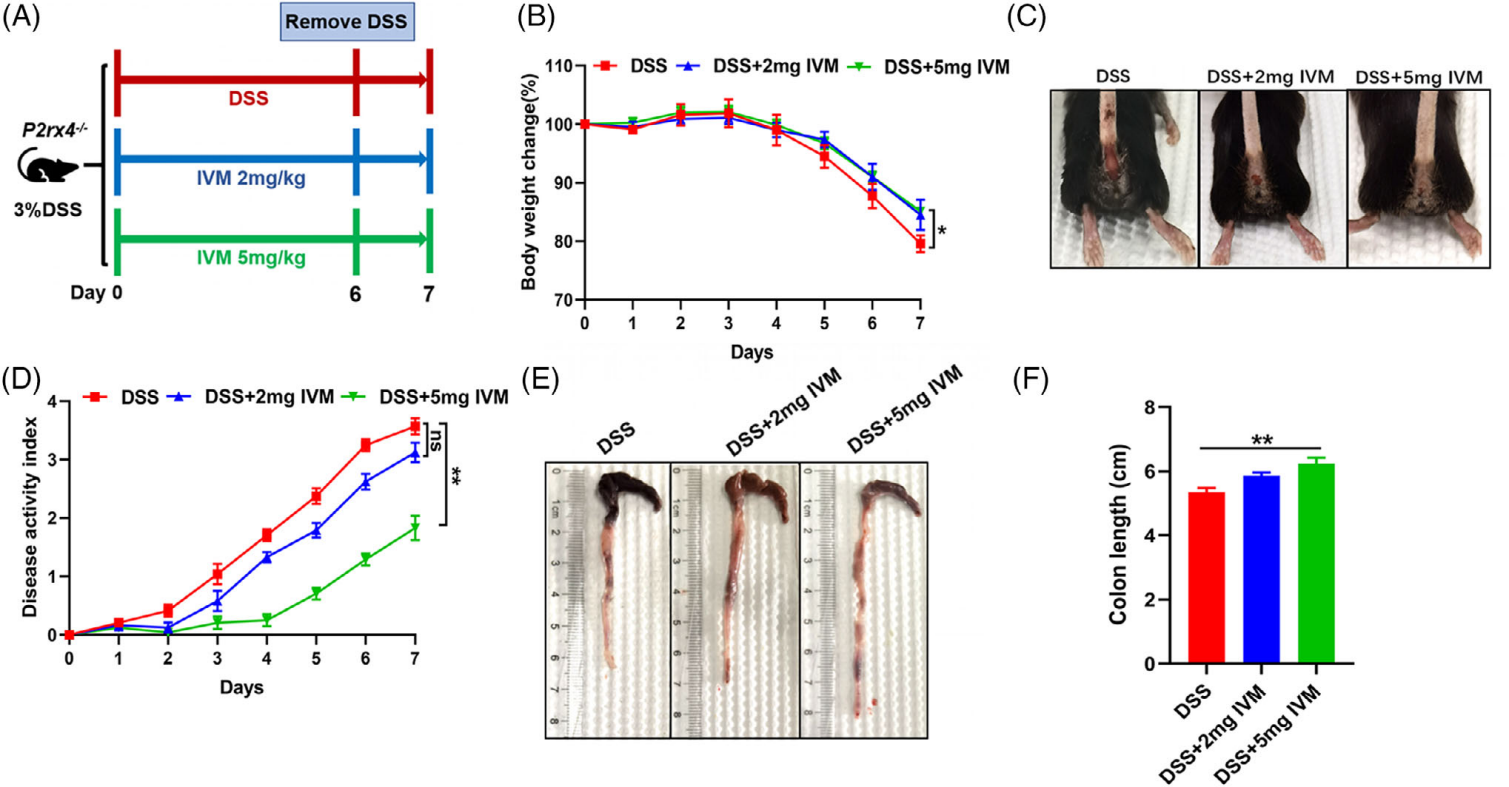
Ivermectin protects dextran sodium sulphate (DSS)‐induced colitis in *P2rx4* ^−/−^ mice. (A) Experimental design of the role of IVM in colitis in *P2rx4* ^−/−^ mice. (B) Body weight change. (C) Representative images of bloody diarrhoea. (D) Disease activity index (DAI) score. (E) Representative pictures of the gross colonic appearance. (F) Colon length is shown as a chart. *p*‐values were calculated using a two‐way analysis of variance (ANOVA) test or two‐tailed unpaired Student's t‐test, *p  <  .05; **p  <  .01; ***p < .001. (DSS, *n*  =  8; DSS+2 mg IVM, *n*  =  8; DSS+5 mg IVM, *n*  =  8).

Finally, we gave *P2rx4* ^−/−^ mice ivermectin at doses of 0, 2 and 5 mg/kg to further ascertain whether the anti‐colitis action of ivermectin relies on the P2X4 receptor (Figure 12A). The results showed that only ivermectin at 5 mg/kg, but not 2 mg/kg, relieved the symptoms of colitis such as weight loss, DAI score, and bowel shortening in DSS‐induced *P2rx4* ^−/−^ mice (Figure 12B–F). These findings indicate that the protective effect of ivermectin against colitis is not dependent on the P2X4 receptor.

## 4 DISCUSSION

Maintaining intestinal homeostasis depends on the gut microbiota's harmonious interactions with the host immunity.^30^ P2X4 is a vital P2 receptor family member that facilitates communication and interaction between the host's immunities and commensal gut microbiota. In this study, we focused on the potential relationship between the P2X4 receptor and gut microbiota in colitis. Our findings suggest that P2X4 receptor inactivation significantly impacts gut microbiota's structure and composition, causing a shift toward a proinflammatory structure that increases colitis susceptibility, indicating that the P2X4 receptor may be a crucial regulator and custodian of intestinal homeostasis. According to the statement of the GSE22307 data provider,^31^ gene expression in the colon alters over time due to the progressive nature of IBD development. The P2X4 receptor changes over time with DSS, likely due to the development of inflammation and subsequent damage to epithelial cells. Indeed, the polarized transport of ions and fluids is one of the primary roles of epithelial cells. This process is regulated by ATP (and other nucleotides) and involves purinergic P2 receptors of the P2X and P2Y subtypes.^9^ Therefore, we speculate that the decrease of P2X4 receptor expression with prolonged exposure to DSS is likely a consequence of the combination of inflammatory progression and epithelial cell damage.

Many factors, including host genetics, disruption of the intestinal barrier, and a dysregulated immune response, contribute to the pathogenesis of a variety of diseases.^32^, ^33^ The P2X4 receptor is identified to exist in various types of epithelial tissues and endothelial cells.^9^ Extracellular ATP is a proinflammatory molecule and the P2X4 receptor can respond to the increased ATP release in response to inflammation.^15^ In this study, it was observed that *P2rx4^−/−^* mice were more prone to colitis with a shorter colon than DSS‐treated WT mice (a reduction of ∼35% for *P2rx4^−/−^* mice and ∼20% for WT mice). To optimize energy gain, the body can adjust the size and shape of the digestive system to react to both the external environment and its inner needs.^34^ The intestinal tract is a complex, multi‐level structure and a change in volume in the digestive tract can be attributed to an altered length. Organizational changes are mainly observed through alterations in the number of microvilli, the thickness of the serous membrane, and other organizational structures.^35^ Therefore, the length index is possibly not descriptive enough when studying the flexible tissue of the intestine. Transmission electron microscopy could be utilized to further observe the changes in mouse microvilli in future experiments. Zabala et al.^36^ reported that P2X4 receptor signaling blockade exacerbated experimental autoimmune encephalomyelitis (EAE) in mice, suggesting that the P2X4 receptor has a similar effect on the two autoimmune diseases. Increasing evidence of a close connection between the brain and the gut has given rise to a new field of research known as the brain‐gut axis,^37^ yet its effects on the P2X4 receptor remain unclear and need to be further investigated.

Further investigation revealed that the worsening colitis caused by *P2rx4* gene ablation may be related to an impaired intestinal mucosal barrier and an altered inflammatory cytokine profile. It is well‐understood that IBD is primarily initiated by a dysregulated mucosal immunity against luminal bacteria.^38^, ^39^, ^40^ Previous studies have proven that bacteria in the gut lumen can result in both acute and chronic intestinal inflammation in patients with IBD.^41^, ^42^ In this study, we found that *P2rx4^−/−^* mice had significantly increased intestinal permeability compared to WT mice given DSS. DSS‐induced *P2rx4^−/−^* mice showed increased translocation of *E. coli* or *E. gallinarum* to mesenteric and inguinal lymph nodes. These findings suggest that the P2X4 receptor is crucial in regulating bacterial translocation and the gut response to injury. Therefore, more experiments are needed to reveal the specific cellular and intercellular mechanisms by which the P2X4 receptor limits bacterial translocation and inflammation.

*P2rx4^−/−^* mice induced by DSS had significantly high levels of ERK, JNK and p38‐MAPK phosphorylation in the colon than DSS‐treated WT mice. Consistent with the phosphorylated MAPK‐related proteins, there were higher concentrations of proinflammatory cytokines in the colon and plasma. It is well known that MAPK signals are activated by an inflammatory signal. Furthermore, intracellular calcium and p38MAPK signal are two other common intracellular messengers that act downstream of the P2X4 receptor, triggering specific cellular functions depending on the cell type that expresses the receptor.^28^ In our study, this finding was further confirmed. However, the specific interaction between the MAPK signalling pathway and the P2X4 receptor is not yet clear. Mapping the entire signaling pathway mediating the effect of the P2X4 receptor is a crucial avenue for future study.

Trillions of different and complex bacteria inhabit the gastrointestinal tract, serving as crucial participants in energy metabolism and exposing the host's vulnerability to various intestinal disorders and diseases.^43^, ^44^ According to studies on the pathophysiology of IBD, a genetically vulnerable host may produce an inappropriate mucosal immune response to commensal microorganisms.^45^, ^46^, ^47^ Previous studies have shown that antibiotic therapy can potentially prevent or mitigate colitis in multiple mouse models of IBD, which is consistent with the results of this study.^48^ Studies suggest that antibiotics can have an anti‐inflammatory effect by maintaining intestinal barrier function via the pregnane X receptor.^49^ Furthermore, antibiotics are capable of replenishing the beneficial bacteria in the gut by reducing the number of bacteria and altering the composition of gut microbiota.^50^ Proteobacteria can be used as a dysbiosis microbial marker in the gut microbiota.^51^ Previous studies have demonstrated that short‐term unstable gut microbiota, especially communities dominated by Proteobacteria, increases the susceptibility to colitis in mice.^52^, ^53^ We collected mouse cecal contents of mice for 16S rDNA sequencing. Unweighted PCoA analysis revealed that the microbiota of the cecal contents differed significantly between the four groups of WT and *P2rx4^−/−^* mice with or without DSS induction. After DSS induction, the abundance of Proteobacteria was significantly higher in *P2rx4^−/−^* mice than in WT mice, indicating that the P2X4 receptor plays a critical role in gut microbiota stabilization.

Furthermore, after receiving broad‐spectrum antibiotics depriving the gut microbiota, *P2rx4^−/−^* mice and WT mice displayed identical colonic inflammation. Interestingly, consistent with previous reports,^54^, ^55^ antibiotic treatment did improve disease activity, especially in *P2rx4^−/−^* colitis mice. Simultaneously, Cohousing and FMT experiments jointly demonstrated that, after homogenizing the microbial communities of *P2rx4^−/−^* and WT mice, the more severe colitis caused by *P2rx4* gene deletion was no longer observed. These results further support our hypothesis that P2X4 receptor expression in the host contributes to the plasticity of the gut microbiota in colitis and that the interaction between the P2X4 receptor and gut microbiota affect the occurrence and progression of IBD.

Ivermectin belongs to the abamectin class, a group of 16‐membered cyclic macrolides,^56^ first introduced as an animal health repellent. Characterized by its high efficacy, broad spectrum, and low toxicity,^57^ it was later approved for human use as an antiparasitic medication in 1987 with minimal adverse effects.^58^, ^59^, ^60^, ^61^, ^62^ Studies have highlighted its anti‐inflammatory, anti‐toxic, and anti‐tumour properties,^63^, ^64^, ^65^ as well as its potential to act as a positive modulator of the P2X4 receptor,^66^ inhibiting the inflammation seen in acetic acid‐induced colitis and EAE.^36^, ^67^ Csóka et al.^16^ demonstrated that ivermectin improves bacterial control and animal survival in sepsis mice by boosting bacterial death in macrophages. In addition, ivermectin has been found to provide a protective effect on apoptosis both in vitro and in vivo, by inducing autophagy and energy damage via AKT/mTOR signalling.^68^ Our study showed that administration of ivermectin at 5 mg/kg alleviated the severity of IBD, including reducing colonic mucosal inflammation and inflammatory cytokine concentrations, as well as decreasing the expression of phosphorylated ERK, JNK and p38. Ulmann et al.^28^ suggested that stimulation of the P2X4 receptor in macrophages triggers calcium influx and phosphorylation of p38 MAPK. However, conflicting results could be due to various experimental environments.

Purinergic ATP‐gated P2X receptors, particularly the P2X4 receptor, are positively modulated by allostery with ivermectin. It is evident that ivermectin does not independently activate P2X4, but rather modifies the current amplitude via ATP.^69^ The exact mechanism of ivermectin's binding to the P2X4 receptor is unknown, but evidence has indicated that ivermectin at low doses enhances the maximal current amplitude, while ivermectin at high doses retards P2X4 receptor deactivation by maintaining the channel open.^70^ Furthermore, existing studies have mostly focused on ivermectin binding to the P2X4 transmembrane domain to stabilize the open state of the channel, but little is known about whether ivermectin can impact *P2RX4 (P2rx4)* gene expression. We thus carried out a second study in which administering ivermectin to *P2rx4^−/−^* mice to see whether the protective effect of ivermectin on colitis depended on the P2X4 receptor. Surprisingly, we found that *P2rx4* ablation did not impair ivermectin's anti‐colitis effect, indicating that the P2X4 receptor is not involved in this protection. Furthermore, T cells from *P2rx4^−/−^* mice were still responsive to ivermectin inhibition; however, the exact mechanism remains unknown, though potential ligands include glutamate‐gated chloride ion channels or ryanodine receptors.^71^, ^72^ We further hypothesized that the protective effect of ivermectin on colitis was related to the regulation of gut microbiota, finding that the α‐diversity of mice given ivermectin at 5 mg/kg decreased, restoring the composition of the gut microbiota to that of the control groups. In addition, *Allobaculum* enrichment, notably related to worsening colitis in mice,^22^, ^73^ was observed to be reduced through ivermectin administration. Altogether, our findings suggest that ivermectin offers protection against IBD by shifting the composition of the gut microbiota.

In addition to ivermectin, other P2X4 receptor activators such as ATP, α‐β‐Metil‐ATP, β‐γ‐Metil‐ATP and BzATP have been shown to activate the entire P2 receptor family with relatively poor selectivity, so they are rarely used for P2X4 receptor studies.^15^, ^74^ Avermectin and moxetine, two analogous members of the lipophilic substance family to ivermectin, are known to render a heightened impact on the P2X4 receptor.^75^, ^76^ Nevertheless, they are supposed to indirectly influence the P2X4 receptor. It is anticipated that medicinal chemists will synthesize more P2X4 receptor‐specific ligands in the coming years.

## 5 CONCLUSIONS

In conclusion, the present study mainly revealed the effect of the P2X4 receptor on acute colitis, which is associated with maintaining gut microbiota dysbiosis. Thus, we envision utilizing P2X4 receptor agonists or ivermectin as adjuvant treatment drugs to treat IBD. In addition, the interaction and crosstalk between the P2X4 receptor and gut microbiota open up new perspectives for IBD treatment.

## CONFLICT OF INTEREST STATEMENT

The authors declare no conflict of interest.

## FUNDING INFORMATION

This work was financially supported by the National Natural Science Foundation of China (U2004104), the Key Project of Henan Education Committee (21A310005), and the Postgraduate Cultivating Innovation and Quality Improvement Action Plan of Henan University (SYLYC2022138, SYL20060187 and SYL20060189). Figdraw drew graphical abstract.

## Supporting information



Supporting Information
