# Supplementary material for: P2X4 receptor modulates gut inflammation and favours microbial homeostasis in colitis
Source: Clin Transl Med. 2023 Apr 21;13(4):e1227. doi: 10.1002/ctm2.1227 (PMC10122071; doi:10.1002/ctm2.1227)
Supplement: Supplementary file 1 — Supporting Information [file CTM2-13-e1227-s001.docx]

**Table S1.** **Genotype identification of PCR primer sequences.**

| Oligo name | Sequence (5′→3′) | Product length/bp |
| --- | --- | --- |
| Primers 1 |  | 694 |
| HOF1 | GTAAGTAACAATAACGCCATGTGCC |  |
| HOR1 | AATTCCTGTGAAGAGAGGACAGG |  |
| Primers 2 |  | 417 |
| HEF2 | GTAAGTAACAATAACGCCATGTGCC |  |
| HER2 | CCCACACGAACACCCACCTGAG |  |

**
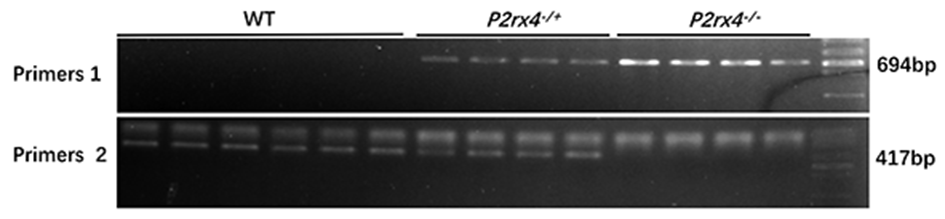
**

**Figure S1. Genotyping results of WT, *P2rx4^-/+^* and *P2rx4^-/-^* mice.** PCR was performed for genotyping gene-edited mice.

**
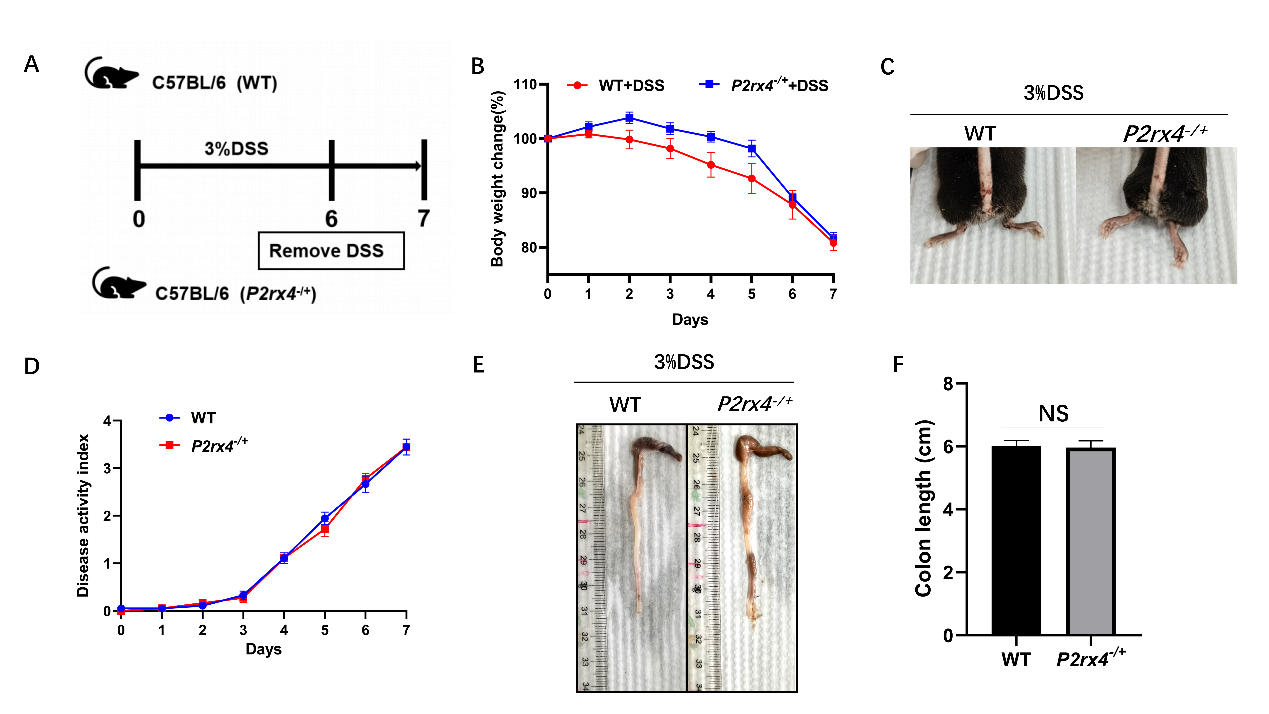
**

**Figure S2. P2RX4 affects DSS-induced colitis in P2rx4^-/+^ and WT mice.** **(A)** Experimental design of DSS-induced colitis. **(B)** Body weight change. **(C)** Representative images of bloody diarrhea. **(D)** DAI score. **(E)** Representative pictures of gross colon appearance. **(F)** Colon length is shown as a chart. **(**WT, *n* = 6; *P2rx4^-/+^*, *n* = 6).


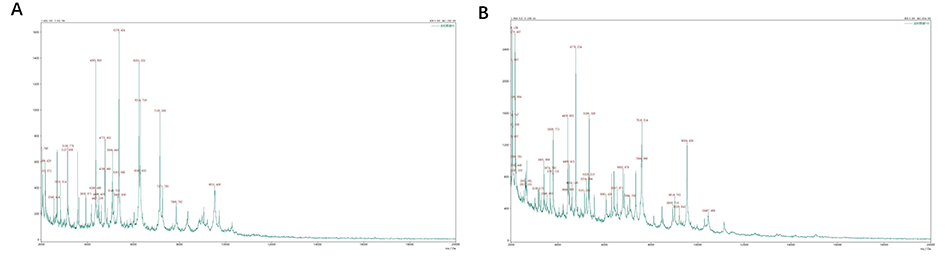


**Figure S3.** **Identification of bacteria by mass spectrometry from the supernatant of mesenteric or inguinal lymph nodes of mice after DSS-induced colitis. (A)** Identification of Escherichia coli by mass spectrometry. **(B)** Identification of Enterococcus gallinarum by mass spectrometry.
